# To what extent do social support and mastery mediate the association between childhood maltreatment and depression? A sequential causal mediation analysis

**DOI:** 10.1017/S2045796022000609

**Published:** 2022-10-20

**Authors:** Y. Y. Su, M. Li, C. D'Arcy, J. Caron, K. O'Donnell, X. Meng

**Affiliations:** 1 Department of Psychiatry, Faculty of Medicine and Health Sciences, McGill University, Montreal, QC, Canada; 2 Douglas Research Centre, Montreal, QC, Canada; 3 School of Public Health, University of Saskatchewan, Saskatoon, SK, Canada; 4 Department of Psychiatry, College of Medicine, University of Saskatchewan, Saskatoon, SK, Canada; 5 Yale Child Study Center & Department of Obstetrics Gynecology & Reproductive Sciences, Yale School of Medicine, Yale University, New Haven, CT, USA; 6 Child & Brain Development Program, CIFAR, Toronto, ON, Canada

**Keywords:** Childhood maltreatment, major depression, mastery, prospective study, social support

## Abstract

**Aims:**

This study aimed to examine the independent roles of various childhood maltreatment (CM) subtypes in the development of depression; quantify the joint mediation effect of social support and mastery in the association between subtypes of CM and depression and examine the additional contribution of mastery beyond the effect that is operating through social support to this relationship.

**Methods:**

Data analysed were from the Zone d’Épidémiologie Psychiatrique du Sud-Ouest de Montréal, an ongoing longitudinal population-based study. In total, 1351 participants with complete information on the studied variables were included. The propensity score matching and inverse-probability weighted regression adjustment estimation methods were used to minimise the potential confounding in the relationship between CM and major depression. We then used inverse odds ratio-weighted estimation to estimate the direct effects of maltreatment and indirect effects of social support and mastery.

**Results:**

We found that exposures to all maltreatment subtypes increased the risk of subsequent depression. The joint mediating effect of social support and mastery explained 37.63–46.97% of the association between different maltreatment subtypes and depression. The contribution of these two mediators differed by maltreatment subtypes, with social support being the major contributor to the mediating effect.

**Conclusions:**

The findings of the study not only provide scientific evidence on the importance of psychosocial attributes in the development of major depression but also suggest that prevention and invention strategies should focus on these psychosocial attributes to effectively break the vicious cycle of CM on major depression.

## Introduction

Major depression is a highly prevalent psychiatric disorder worldwide with a 12-month prevalence of 11% and a lifetime prevalence of up to 21% (Gutiérrez-Rojas *et al*., 2020). It affects all aspects of life, including physical and psychological well-being and social functioning (Knoll and MacLennan, 2017). Because of its profound health impact and economic burden, it ranks as the second highest cause of years lost due to disability (Vos *et al*., 2015).

Childhood maltreatment (CM) is one of the most important risk factors. Accumulating evidence has shown that exposure to CM predicts the elevated risk of subsequent major depression (Nelson *et al*., 2017). CM refers to the abuse and neglect that occurs to children before 18 years of age which consists of several subtypes, including physical abuse, emotional abuse, sexual abuse, physical neglect and emotional neglect (Sedlak *et al*., 2010). CM subtypes differentially influence the development of aetiological pathways in subsequent depression. A recent meta-analysis discovered that emotional abuse had a stronger association with the later development of major depression than other CM subtypes (Infurna *et al*., 2016). Similarly, a meta-analysis of 190 studies consisting of 68 830 individuals also found that emotional abuse and emotional neglect had the strongest associations with depression (Humphreys *et al*., 2020). Emotional abuse has been viewed as the ‘core component’ of all CM subtypes and is more prevalent than physical abuse or sexual abuse (Festinger and Baker, 2010). Those exposed to emotional abuse were at a higher risk of developing a more negative self-model and negative self-schemas, thus becoming more vulnerable to major depression (Rose and Abramson, 1992). However, Nelson and colleagues in their meta-analysis found no significant differences between CM subtypes and the risk of depression (Nelson *et al*., 2017). Therefore, it is crucial to verify whether CM subtypes have a uniform impact on depression or it acts differently in depression when different subtypes are examined.

Noteworthily, not all individuals exposed to CM would develop depression in later life. As suggested by the diathesis-stress theory, both biological and psychosocial factors are involved in the psychological processes of CM and may explain why some end up with depression and some do not (Arnau-Soler *et al*., 2018). Genetic variations could explain up to 10% of the population variation in the risk of depression (Howard *et al*., 2019). Many genetic variations are having small effects as polygenic risk score (PRS) is more widely used to quantify the cumulative effect of single polymorphisms (Dudbridge, 2013). Howard and colleagues analysed a total of 807 553 individuals from three large genome-wide association studies (GWASs) of major depression and discovered 102 independent variants, mapping on a total of 269 genes, and 115 gene sets associated with major depression (Howard *et al*., 2019). They estimated the single-nucleotide polymorphism (SNP)-based heritability of major depression was 0.089 indicating a significant genetic component in major depression. Thus, the contribution of genetic factors to the aetiology of major depression is not neglectable.

In addition to genetic prepositions, psychosocial factors are assumed to play a prominent role in depression. Social support plays a critical role in the association between CM and depression (Sperry and Widom, 2013). Social support refers to the provision of psychological and material resources used to cope with stress (Chu *et al*., 2010). It acts as a key external resource and is one of the principal psychosocial factors in protecting against the adverse effect of CM (Su *et al*., 2020). The stress-buffering model suggests that social support impacts health-related outcomes through its influence on the appraisal of the stressful situation (Cohen and Wills, 1985). Longitudinal studies have confirmed the mediation effects of social support on the association between self-reported maltreatment and major depression (Salazar *et al*., 2011).

Mastery is an internal resource for individuals to cope with the long-term effects of CM and is found to be protective against major depression (King *et al*., 2015). A sense of mastery is the ability of an individual to control important aspects of life. It helps individuals to perceive higher control over stressful situation and turns to social networks to help reduce the negative effects of stressors (Infurna *et al*., 2015). An integral component of the stress process model focuses on psychological resources, such as the sense of mastery, which is theorised to modify the impact of both the primary and contextual stressors (Pearlin *et al*., 1990). In addition, psychological theories have focused on the potential significance of mastery in the aetiology of major depression (Carver and Gaines, 1987). There has been extensive evidence on higher levels of mastery are associated with lower depressive symptoms over time (Steunenberg *et al*., 2007). The literature has suggested the mediating role of mastery in the association between CM and major depression (Hovens *et al*., 2016). In addition, according to the transactional model of stress, internal and external resources are influential psychosocial factors in determining an individual's stress appraisals (Lazarus and Folkman, 1984). Social support and mastery are also closely interconnected with each other. Research has shown that individuals with more social support are more likely to have higher personal control (Crowe *et al*., 2016). In turn, individuals with a high level of mastery generally have larger available support networks than those with lower mastery (Hansson *et al*., 1984).

Although the associations between CM and depression and the potential roles of social support and mastery involved in these associations have been separately examined, little is known about how social support and mastery are simultaneously involved in these associations. In addition, even less is conducted to comprehensively investigate whether their mediating roles would be different for different CM subtypes. Most studies are cross-sectional, which have methodological issues in terms of the likelihood of having biased estimates and causal inference. Large-scale longitudinal studies are needed to articulate how and to what extent social support and mastery mediate the association between CM and depression. We are not aware of any study that has reported this association using large longitudinal data. An in-depth understanding of mediation is critical for effectively intervening in the negative sequelae of maltreatment in depression.

**Fig. 1.**
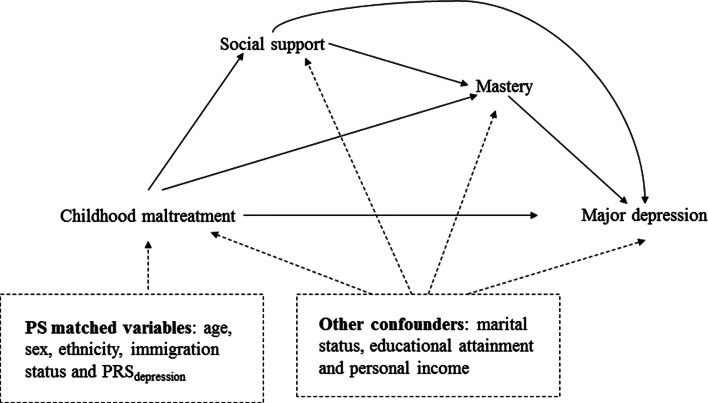
Causal diagram for the hypothesised effects of childhood maltreatment on major depression through social support and mastery.

The present study aimed to (1) examine the independent roles of multiple maltreatment subtypes in depression; (2) quantify the joint mediation effect of social support and mastery in the association between different CM subtypes and depression; and, (3) examine the additional contribution of mastery beyond the effect that is operating through social support to this relationship. Figure 1 illustrates the conceptual framework of the present study. We hypothesised that: (1) all CM subtypes would increase the risk of subsequent depression and (2) the mediation effects of social support and mastery in these relations would vary between CM subtypes.

## Methods

### Study cohort

Data analysed were from the Montreal South-West Longitudinal Catchment Area Study-Zone d’Épidémiologie Psychiatrique du Sud-Ouest de Montréal (ZEPSOM). The ZEPSOM is a large-scale, longitudinal population-based cohort from southwest Montreal, Canada. The initial cohort sample consisting of 2434 individuals was recruited between June 2007 and November 2008. From July 2009 to November 2010, 1823 participants responded to the interview with a retention rate of 75.0% at wave 2. In total, 1303 of the initial participants were followed to the wave 3 (between July 2012 and July 2013). At the same time, a second cohort comprising 1029 participants for attrition was recruited in wave 3 in order to maintain a representative sample of the population. Additional 1037 participants were enrolled and interviewed for wave 4 of which 534 remained in the study at 2-year intervals followed (January 2014 until April 2015). The cooperation rate of this longitudinal cohort was 71.6% for wave 3 and 80.2% for wave 4. A total of 1406 participants were recruited in wave 5 (conducted in 2017–2018) with an attrition rate of 24.8%.

**Fig. 2.**
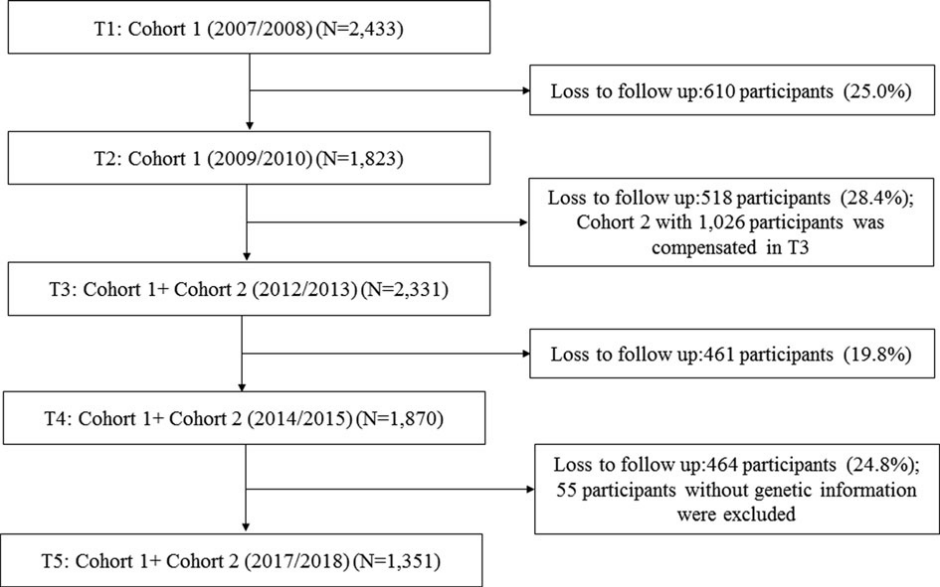
Flow chart of the study cohort.

The present study included those ZEPSOM participants who completed waves 3–5 and had the information on the studied variables (major depression, CM, social support, and mastery). Figure 2 illustrates the process of the study sample selection. A total of 1351 participants, who have completed the survey on CM (wave 5), social support (wave 3), mastery (wave 4), major depression (wave 4), and other covariates (wave 3), were included in the present study.

### Measures

#### Childhood maltreatment

CM was assessed using the Childhood Trauma Questionnaire (CTQ-28) (Bernstein and Fink, 1998). It is a self-reported instrument that assesses five subtypes of CM during childhood and adolescence: emotional abuse, emotional neglect, physical abuse, physical neglect, and sexual abuse. Respondents rate each item on a 5-point Likert scale (‘never true’ to ‘very often true’). The cut-off points for each CM subtype used in the present study were following the suggestion of Bernstein and Fink (Bernstein *et al*., 1998). The cut-off points for the presence of a specific CM subtype were: the total score of physical abuse ⩾8; the total score of sexual abuse ⩾6; the total score of emotional abuse ⩾9; the total score of physical neglect ⩾8;or the total score of emotional neglect ⩾10.

#### Major depression

Major depression was assessed based on the WHO's World Mental Health (WMH) 2000 project (WMH-CIDI) (Kishore *et al*., 1999). The WMH-CIDI is a fully structured diagnostic interview assessing major depression using the definitions and criteria of the Diagnostic and Statistical Manual of Mental Disorders, 4th edition (DSM-IV) and the International Statistical Classification of Diseases and Related Health Problems, 10th revision (ICD-10) (American Psychiatric Association, 2000; World Health Organization, 1992).

#### Social support

Social support was assessed with the 24-item Social Provision Scale (SPS) (Cutrona and Russell, 1987). It consists of six social provisions including attachment, social integration, reassurance of worth, reliable alliance, guidance, and opportunity for nurturance. Each provision was assessed by four items, and items are scored along a 4-point Likert scale (1 = strongly disagree to 4 = strongly agree). The Cronbach's alpha value of SPS in this study was 0.91.

#### Mastery

Mastery was assessed using the Pearlin's Mastery Scale, a 7-item measure with a 5-point scale to assess personal mastery (Pearlin and Schooler, 1978). Response options ranged from 1 (strongly agree) to 5 (strongly disagree). The scale has been used to assess the control of one's life and has been widely used in health research. Its Cronbach's alpha value was 0.72.

#### Other variables included in the present study

In the present study, we also considered two sets of covariates. The following variables, including PRS_depression,_ age, sex, ethnicity and immigration status were treated as matching variables for propensity score analysis. PRS_depression_ was generated using the GWAS summary statistics of all SNPs (*p*-value threshold at 0.001) from the Psychiatric Genomics Consortium (PGC). PRS_depression_ was calculated by summing the number of risk alleles weighted by their effect size for each ZEPSOM participant. Another set of variables including marital status, educational attainment and personal income, was also adjusted in all the analyses.

### Statistical analysis

#### Propensity score matched the study sample on CM

To investigate the likelihood of major depression for those exposed to and not exposed to CM, we used propensity score analysis to reduce bias. First, a three-step propensity score matching (PSM) combined with the inverse probability-weighted regression-adjustment (IPWRA) analyses were conducted to investigate the effects of CM on depression, following the process recommended by Guo and Fraser (2014). The first step was to obtain the propensity score, and the second step was to match each CM victim with a non-CM victim on their estimated propensity scores. Next, the estimated propensity score obtained for each participant was used to determine whether the scores had a common-support region, which would permit 3-1 nearest-neighbour matching. Then, the standardised mean difference was used to measure the balance between maltreated groups and non-maltreated groups on baseline characteristics after matching. Online Supplementary Fig. S1 shows the pipeline of the PSM process. We also employed post-matching statistical techniques to assess the quality of matching. The treatment effects were estimated using IPWRA with corrected standard errors. It combines augmented inverse probability weighting of covariates and regression variable adjustment to concurrently estimate the outcome parameters.

#### Mediation roles of social support and mastery in the relationship between CM and major depression

The sequential mediation analysis was then conducted with the inverse odds ratio-weighted (IOW) estimation to estimate the direct effects of maltreatment and indirect effects of social support and mastery in the matched sample. The IOW approach can provide direct and indirect effect estimates in the presence of nonlinearities and interactions. Additionally, the IOW approach does not need to specify exposure–mediation interactions therefore fewer modelling assumptions are required (Nguyen *et al*., 2016). Sensitivity analysis was performed to compare the current results with the analyses for those participants with incident depression only. Multiple imputations (with five imputed datasets) were conducted to estimate the average natural direct effect (NDE) and average natural indirect effect (NIE). Bootstrapping was performed based on 1000 replications to derive 95% confidence intervals (CIs) for all mediation parameters after adjusting for marital status, educational attainment and personal income. To ease the interpretations, we presented risk ratios (RRs) and the proportion mediated by mediators using the formula:
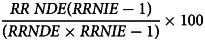


All analyses were conducted in Stata, version 15.

## Results

### Characteristics of the study sample

The present study included a total of 1351 participants, with 853 (63.1%) females. The mean age of the study cohort was 50.6 ± 13.8 years and one-thirds aged between 45 and 60 years. The majority (80.9%) of the respondents were non-immigrants. In total, 43.7% of respondents were single and over half (65.3%) indicated that they had completed a post-secondary degree. Mean household income was $34 175 (s.d. = 38 872), and more than half of the sample reported earning $30 000–60 000 annually. Characteristics of the study population are shown in online Supplementary Table S1. The prevalence of emotional abuse and emotional neglect was 28.1 and 46.7%, respectively. The prevalence of physical abuse and physical neglect was 21.1 and 22.9%, respectively. The prevalence of sexual abuse was 29.6%. In addition, 23.4% of the current sample reported having major depression. The correlations among the studied variables in this study are shown in Table 1. All the studied variables were significantly correlated (*p* < 0.05). Different CM subtypes were negatively correlated with social support and mastery but were positively correlated with major depression. Social support and mastery were negatively correlated with major depression.

**Table 1.** Correlations among subtypes of CM, social support, mastery and major depression

Variables	1	2	3	4	5	6	7	8
1. Physical abuse	1							
2. Emotional abuse	0.47*	1						
3. Sexual abuse	0.32*	0.37*	1					
4. Physical neglect	0.38*	0.34*	0.25*	1				
5. Emotional neglect	0.33*	0.45*	0.23*	0.40*	1			
6. Social support	−0.19*	−0.17*	−0.09*	−0.22*	−0.20*	1		
7. Mastery	−0.12*	−0.17*	−0.14*	−0.16*	−0.18*	0.38*	1	
8. Major depression	0.12*	0.17*	0.09*	0.12*	0.10*	−0.17*	−0.20*	1

Note: *p < 0.05.

### Analysis of the propensity matched sample

Online Supplementary Tables S2–S6 present adjusted and unadjusted means for the matching variables in the current study sample. We compared the observed effect sizes before and after the PSM process to examine whether the matching process reduced the standardised differences between maltreatment and non-maltreatment groups. Non-significant results of the independent sample t-test of each matching variable between maltreatment and non-maltreatment groups after the PSM procedure suggested the matching was appropriately done. Maltreatment and non-maltreatment groups were well balanced for each matching variable for different CM subtypes. Online Supplementary Fig. S2 shows the distribution of the propensity scores of maltreated respondents and non-maltreated respondents.

The average treatment effect on treated (ATE) was estimated with the IPWRA based on the matched sample. Table 2 provides ATEs for different CM subtypes in major depression. All types of CM significantly increased the risk of major depression. For instance, the average difference in the probability of having major depression for individuals exposed to emotional abuse compared to those without emotional abuse was 0.162, indicating that emotional abuse increased 16.2% of the chance of having depression compared to non-emotional abuse. The probability of having major depression was 14.0% higher for individuals exposed to physical neglect compared to non-physical neglect ones, followed by physical abuse (12.3%), sexual abuse (8.3%) and emotional neglect (7.5%).

**Table 2.** ATEs of different maltreatment subtypes on major depression

Maltreatment types	Major depression			
	ATE	Robust s.e.	*p* value	95% CI
Emotional abuse	0.162	0.031	<0.001	0.103–0.222
Physical abuse	0.123	0.035	<0.001	0.054–0.193
Sexual abuse	0.083	0.031	0.007	0.022–0.144
Emotional neglect	0.075	0.027	0.005	0.022–0.128
Physical neglect	0.140	0.033	<0.001	0.076–0.205

ATE, average treatment effects; s.e., standard error; CI, confidence interval.

### Results of casual mediation analyses after PSM

The casual mediation analysis was conducted for each CM subtype based on the matched sample. Table 3 summarises the causal mediation analysis results for each CM subtype. The total effects of different CM subtypes on major depression remained significant after considering the individual mediator and two mediators simultaneously, as well as other covariates (marital status, educational attainment and income). The proportion of the total effect of CM on major depression mediated by both social support and mastery ranged from 37.63 to 46.97%. Compared to mastery, social support demonstrated a greater mediating role in the associations of different CM subtypes and major depression. For instance, 46.97% of the total effect of emotional abuse on depression was mediated by social support and mastery (RR_natural indirect effect_ = 1.27; 95% CI: 1.16–1.40), and social support itself contributed to 31.89% of this total effect (RR_natural indirect effect_ = 1.17; 95% CI: 1.08–1.26) whereas mastery explained 15.08% of this total effect. This phenomenon is consistent for all different CM subtypes, with social support mediating 24.35–37.94% of the association between CM and major depression. We observed similar results in the sensitivity analyses (see online Supplementary Table S7).

**Table 3.** Total, direct and indirect effect of the association between CM and major depression

Maltreatment	Mediators	TCE	NDE	NIE	Proportion mediated (%)	Additional contribution (%)
		RR (95% CI)	RR (95% CI)	RR (95% CI)		
Emotional abuse	Social support	1.83 (1.53–2.20)	1.57 (1.29–1.90)	1.17 (1.08–1.26)	31.89	–
	+ Mastery	1.83 (1.53–2.20)	1.44 (1.16–1.79)	1.27 (1.16–1.40)	46.97	15.08
Physical abuse	Social support	1.61 (1.32–1.97)	1.38 (1.09–1.74)	1.17 (1.06–1.28)	37.94	–
	+ Mastery	1.61 (1.32–1.97)	1.36(1.05–1.74)	1.19 (1.08–1.31)	42.05	4.11
Sexual abuse	Social support	1.44 (1.19–1.76)	1.30 (1.07–1.58)	1.11 (1.05–1.17)	32.11	–
	+ Mastery	1.44 (1.19–1.76)	1.25 (1.01–1.54)	1.16 (1.08–1.25)	44.75	12.64
Emotional neglect	Social support	1.38 (1.12–1.70)	1.28 (1.07–1.51)	1.08 (1.02–1.15)	27.40	–
	+ Mastery	1.38 (1.12–1.70)	1.21 (0.97–1.50)	1.14 (1.06–1.24)	45.84	18.44
Physical neglect	Social support	1.60 (1.32–1.95)	1.46 (1.17–1.81)	1.10 (1.01–1.20)	24.35	–
	+ Mastery	1.60 (1.32–1.95)	1.38 (1.11–1.70)	1.16 (1.02–1.33)	37.63	13.28

NDE, natural direct effect; NIE, natural indirect effect; TCE, total causal effect; RR, risk ratio; CI, confidence interval.

## Discussion

This present study provides one of the first pieces of evidence on the mediating effects of social support and mastery in the associations between different CM subtypes and major depression in a longitudinal cohort. We used the PSM combined with the sequential casual mediation analysis approach which takes full advantage of longitudinal data by bringing analytic models in line with theorising through developing process-based models of a causal relationship. All different CM subtypes were significantly associated with a higher risk of major depression. Both social support and mastery acted as important mediators in these associations, with social support being the major mediator. The findings of the study not only provide scientific evidence on the importance of psychosocial attributes in the development of major depression but also suggest that prevention and invention strategies should focus on these psychosocial attributes to effectively break the vicious cycle of CM on major depression.

Our findings are consistent with previous research on the relationships between CM and depressive symptoms in adulthood (Gallo *et al*., 2018). Our results also extend the previous literature by identifying differential effects of individual types of CM on major depression. Exposure to emotional abuse was the most important risk factor for major depression. This is in line with the results of a meta-analysis indicating that emotional abuse was shown to be most closely related to depression severity (Nelson *et al*., 2017). Likewise, emotional abuse exhibited the strongest correlation with depression even after controlling for other types of maltreatment in a northern California community sample (Caldwell *et al*., 2011). According to the hopelessness theory of depression proposed by Rose and Abramson, emotional abuse makes individuals particularly vulnerable to developing negative cognitive coping skills, which in turn increase the risk for depression (Rose and Abramson, 1992).

As expected, social support and mastery were identified as important mediators in the associations between CM subtypes and major depression. In line with the stress-buffering model (Lazarus and Folkman, 1984), our results suggested that social support buffered the effects of CM on subsequent depression. Empirical studies have confirmed the protective role of social support in inhibiting and mitigating the long-term adverse consequences of CM on major depression (Negriff *et al*., 2019). Sperry and Widom (2013) found that social support mediated the relationship between childhood abuse and subsequent mental health outcomes, including depression.

Our results did find that mastery mediated the association between CM and depression. Consistently, a high level of mastery attenuated the association between childhood abuse and depressive symptoms (King *et al*., 2015). Likewise, a Netherlands longitudinal cohort study of 2981 adults also found the mediating role of mastery in the association between CM and major depression in the 4-year follow-up (Hovens *et al*., 2016).

Furthermore, the joint mediating roles of social support and mastery were observed in the association of CM and depression. This could be partially explained by the resources theory, which argues that people who have access to more resources, are not only more resilient in stressful situations but also know how to adaptively cope with stressful situations (Hobfoll, 1988). Kim and colleagues in their cross-sectional study found that higher levels of social support and mastery significantly mediated the negative effects of general life stress on major depression (Kim *et al*., 2005). Our findings also revealed a stronger mediating effect of social support than mastery. The stress-buffering effect of social support has been shown to be the most effective factor when the support met the needs of individuals with stressors (Uchino, 2009). Another explanation of the stronger effect of social support in the association of maltreatment and depression may be related to the high availability of social support promoting programmes and strategies (Ozbay *et al*., 2007). Internal resources of individuals may vary from one to another. It is possible that only certain psychological resources have a conspicuous influence on the association between maltreatment and depression (Lunau *et al*., 2018).

### Strengths and limitations

This study is one of a few studies examining the underlying mechanisms of social support and mastery in the association between CM and depression in a longitudinal cohort. It adopts strong methodological approaches, including longitudinal study design as well as the use of the PSM method to reduce the potential confounders involved in the relationship between maltreatment and depression, and sensitivity analysis to compare the results.

There are several limitations to be noted. First, CM was retrospectively measured by a self-reported questionnaire that may cause measurement bias leading to spurious results. In addition, although CTQ was widely used to measure adverse childhood experiences and produced valid results it is difficult to identify unique exposure to specific CM subtypes as they often co-occur. Second, the present study did not have access to other dimensions of CM, such as duration, severity, initial age of the abuse, perpetrator and the frequency of the abuse. Thus, future research should fully consider the multidimensional nature of CM and evaluate other dimensions of CM with different measures which contain sections to assess the presence, frequency, duration and severity of CM subtypes. Third, mastery and major depression were measured at the same time. It is possible that exposure to CM could cause major depression then influences mastery. However, mastery is a stable personality disposition that once has been formed at an early life stage is more likely to remain stable unless interventions of mastery are taken (Rodríguez-Cifuentes *et al*., 2020). Because previous studies have supported the pathway from CM to social support and mastery, which then led to poor mental health outcomes (Hovens *et al*., 2016; Negriff *et al*., 2019). The present study was inspired by this conceptual framework. Finally, loss to follow-up in different waves of the current longitudinal study could present a risk to the internal validity of the study findings.

## Conclusion

The present study supported the simultaneously mediating effects of social support and mastery in the associations between different CM subtypes and major depression and discovered differential mediations in the associations between CM subtypes and depression in a longitudinal cohort. Our results provide robust evidence to suggest targeted and selective prevention efforts on these two mediators to break down the vicious cycle of CM on major depression. Intervention programmes and strategies designed to enhance social networks in the community and mastery of stress intervention at both school and societal levels that effectively mobilise personal resources should be encouraged.

## Data Availability

The ZEPSOM data are not currently freely available to researchers in general due to ethical and data management requirements. Interested researchers can directly contact the Research Team at: Zepsom.Coordo@douglas.mcgill.ca.

## Supplementary material

For supplementary material accompanying this paper visit https://doi.org/10.1017/S2045796022000609. click here to view supplementary material
