# Supplementary material for: To what extent do social support and mastery mediate the association between childhood maltreatment and depression? A sequential causal mediation analysis
Source: Epidemiol Psychiatr Sci. 2022 Oct 20;31:e77. doi: 10.1017/S2045796022000609 (PMC9677445; doi:10.1017/S2045796022000609)
Supplement: Supplementary file 1 [file S2045796022000609sup001.docx]

| **Table S1**. Sociodemographic characteristics of the study cohort (N=1351). | | |  | |
| --- | --- | --- | --- | --- |
| Sociodemographic characteristics | N | Percentage | |  |
| Sex |  |  | |  |
| Male | 498 | 36.9% | |  |
| Female | 853 | 63.1% | |  |
| Age |  |  | |  |
| 18-30 years | 135 | 10.0% | |  |
| 30-45 years | 364 | 26.9% | |  |
| 45-60 years | 470 | 34.8% | |  |
| 60 years and above | 382 | 28.3% | |  |
| Marital status |  |  | |  |
| Single | 590 | 43.7% | |  |
| Married/Common-law | 502 | 37.2% | |  |
| Separated/Divorced/Widowed | 259 | 19.2% | |  |
| Ethnicity |  |  | |  |
| White | 1176 | 87.0% | |  |
| Non-white | 175 | 13.0% | |  |
| Immigration status |  |  | |  |
| Yes | 250 | 19.1% | |  |
| No | 1060 | 80.9% | |  |
| Educational attainment |  |  | |  |
| Less than secondary education | 92 | 6.8% | |  |
| Secondary education | 145 | 10.8% | |  |
| Some post-secondary | 232 | 17.2% | |  |
| Post-secondary degree | 882 | 65.3% | |  |
| Income |  |  | |  |
| Less than $10,000 | 178 | 13.2% | |  |
| $10,000 to $30,000 | 257 | 19.0% | |  |
| $30,000 to $60,000 | 754 | 55.8% | |  |
| $60,000 or more | 162 | 12.0% | |  |
|  |  |  | |  |
|  |  |  | |  |
|  |  |  | |  |

| **Table S2**. Estimates on the propensity score matching for emotional abuse. | | | | | |  |
| --- | --- | --- | --- | --- | --- | --- |
| Predictor variables | Sample | Mean | | Std Diff | t-test | |
|  |  | Maltreated | Non-maltreated |  | t | p-value |
| Age | Unmatched | 50.076 | 50.711 | -4.6 | -0.76 | 0.449 |
|  | Matched | 50.076 | 49.267 | 5.9 | 0.81 | 0.420 |
| Female | Unmatched | 0.739 | 0.586 | 32.8 | 5.28 | 0.001 |
|  | Matched | 0.739 | 0.734 | 1.1 | 0.16 | 0.869 |
| Ethnicity | Unmatched | 0.124 | 0.129 | -1.7 | -0.29 | 0.775 |
|  | Matched | 0.124 | 0.130 | -1.8 | -0.25 | 0.800 |
| Immigration status | Unmatched | 0.845 | 0.804 | 10.7 | 1.72 | 0.085 |
|  | Matched | 0.845 | 0.839 | 1.4 | 0.20 | 0.843 |
| Polygenetic risk score | Unmatched | -0.003 | -0.003 | 12.7 | 2.12 | 0.034 |
|  | Matched | -0.003 | -0.002 | -7.7 | -1.04 | 0.298 |

| **Table S3**. Estimates on the propensity score matching for physical abuse. | | | | | |  |
| --- | --- | --- | --- | --- | --- | --- |
| Predictor variables | Sample | Mean | | Std Diff | t-test | |
|  |  | Maltreated | Non-maltreated |  | t | p-value |
| Age | Unmatched | 51.733 | 50.140 | 11.9 | 1.72 | 0.085 |
|  | Matched | 51.733 | 52.674 | -7.0 | -0.85 | 0.394 |
| Female | Unmatched | 0.630 | 0.629 | 0.2 | 0.003 | 0.974 |
|  | Matched | 0.630 | 0.649 | -3.9 | -0.47 | 0.640 |
| Ethnicity | Unmatched | 0.157 | 0.121 | 10.2 | 1.57 | 0.116 |
|  | Matched | 0.157 | 0.148 | 2.4 | 0.27 | 0.785 |
| Immigration status | Unmatched | 0.972 | 0.988 | -11.4 | -1.92 | 0.055 |
|  | Matched | 0.972 | 00974 | -1.7 | -0.17 | 0.863 |
| Polygenetic risk score | Unmatched | -0.002 | -0.003 | 24.2 | 3.64 | 0.000 |
|  | Matched | -0.002 | -0.002 | 3.2 | 0.38 | 0.703 |
|  |  |  |  |  |  |  |
|  |  |  |  |  |  |  |

| **Table S4**. Estimates on the propensity score matching for sexual abuse. | | | | | |  |
| --- | --- | --- | --- | --- | --- | --- |
| Predictor variables | Sample | Mean | | Std Diff | t-test | |
|  |  | Maltreated | Non-maltreated |  | t | p-  value |
| Age | Unmatched | 52.338 | 49.720 | 19.5 | 3.17 | 0.002 |
|  | Matched | 52.338 | 52.190 | 1.1 | 0.16 | 0.877 |
| Female | Unmatched | 0.713 | 0.594 | 25.2 | 4.13 | 0.001 |
|  | Matched | 0.713 | 0.714 | -0.4 | -0.05 | 0.958 |
| Ethnicity | Unmatched | 0.127 | 0.130 | -1.2 | -0.2 | 0.840 |
|  | Matched | 0.127 | 0.142 | -4.8 | -0.66 | 0.510 |
| Immigration status | Unmatched | 0.977 | 0.986 | -6.7 | -1.18 | 0.240 |
|  | Matched | 0.977 | 0.970 | 5.0 | 0.59 | 0.553 |
| Polygenetic risk score | Unmatched | -0.002 | -0.003 | 20.4 | 3.43 | 0.001 |
|  | Matched | -0.002 | -0.003 | 2.6 | 0.37 | 0.709 |

| **Table S5**. Estimates on the propensity score matching for physical neglect. | | | | | |  |
| --- | --- | --- | --- | --- | --- | --- |
| Predictor variables | Sample | Mean | | Std  Diff | t-test | |
|  |  | Maltreated | Non-maltreated |  | t | p-value |
| Age | Unmatched | 51.389 | 49.757 | 11.9 | 2.16 | 0.031 |
|  | Matched | 51.389 | 52.078 | -5.0 | -0.89 | 0.376 |
| Female | Unmatched | 0.654 | 0.610 | 9.3 | 1.68 | 0.092 |
|  | Matched | 0.654 | 0.649 | 1.1 | 0.20 | 0.843 |
| Ethnicity | Unmatched | 0.116 | 0.1427 | -7.9 | -1.43 | 0.152 |
|  | Matched | 0.116 | 0.133 | -5.0 | -0.89 | 0.374 |
| Immigration status | Unmatched | 0.974 | 0.992 | -13.4 | -2.47 | 0.014 |
|  | Matched | 0.974 | 0.970 | 2.9 | 0.40 | 0.687 |
| Polygenetic risk score | Unmatched | -0.003 | -0.003 | 11.2 | 2.03 | 0.042 |
|  | Matched | -0.003 | -0.003 | -0.5 | -0.08 | 0.935 |

| **Table S6**. Estimates on the propensity score matching for emotional neglect. | | | | | |  |
| --- | --- | --- | --- | --- | --- | --- |
| Predictor variables | Sample | Mean | | Std Diff | t-test | |
|  |  | Maltreated | Non-maltreated |  | t | p-value |
| Age | Unmatched | 52.066 | 50.048 | 15.0 | 2.25 | 0.025 |
|  | Matched | 52.066 | 51.916 | 1.1 | 0.14 | 0.889 |
| Female | Unmatched | 0.671 | 0.6139 | 11.9 | 1.81 | 0.07 |
|  | Matched | 0.671 | 0.697 | -5.5 | -0.70 | 0.486 |
| Ethnicity | Unmatched | 0.138 | 0.125 | 3.9 | 0.60 | 0.551 |
|  | Matched | 0.138 | 0.136 | 0.6 | 0.08 | 0.937 |
| Immigration status | Unmatched | 0.974 | 0.986 | -9.0 | -1.51 | 0.130 |
|  | Matched | 0.974 | 0.980 | -4.7 | -0.54 | 0.589 |
| Polygenetic risk score | Unmatched | -0.002 | -0.003 | 17.4 | 2.67 | 0.008 |
|  | Matched | -0.002 | -0.002 | 1.3 | 0.16 | 0.876 |
|  |  |  |  |  |  |  |
|  |  |  |  |  |  |  |

| **Table S7**. Total, direct and indirect effect of the association between childhood maltreatment and incident major depression. | | | | | |
| --- | --- | --- | --- | --- | --- |
| Maltreatment | Mediators | TCE | NDE | NIE |  |
|  |  | RR (95% CI) | RR (95% CI) | RR (95% CI) |  |
| Emotional abuse | Social support | 2.04(1.34, 3.12) | 1.41(0.91, 2.18) | 1.45(1.23, 1.72) |  |
|  | + Mastery | 2.04(1.34, 3.12) | 1.16(0.74, 1.82) | 1.76(1.47, 2.10) |  |
| Physical abuse | Social support | 1.77(1.17, 2.69) | 1.04(0.64, 1.68) | 1.70(1.37, 2.13) |  |
|  | + Mastery | 1.77(1.17, 2.69) | 1.01(0.61, 1.64) | 1.76(1.43, 2.17) |  |
| Sexual abuse | Social support | 1.23(0.83, 1.83) | 0.94(0.63, 1.43) | 1.31(1.16, 1.47) |  |
|  | + Mastery | 1.23(0.83, 1.83) | 0.84(0.52, 1.35) | 1.47(1.24, 1.75) |  |
| Emotional neglect | Social support | 1.64(1.10, 2.42) | 1.19(0.77, 1.85) | 1.38(1.17, 1.61) |  |
|  | + Mastery | 1.64(1.10, 2.42) | 1.03(0.69, 1.55) | 1.58(1.36, 1.83) |  |
| Physical neglect | Social support | 1.92(1.32, 2.81) | 1.24(0.71, 2.16) | 1.55(1.23, 1.96) |  |
|  | + Mastery | 1.92(1.32, 2.81) | 1.06(0.70, 1.61) | 1.81(1.44, 2.29) |  |

Note: NDE: natural direct effect; NIE: natural indirect effect; TCE: total causal effect; RR: risk ratio; CI: confidence interval.


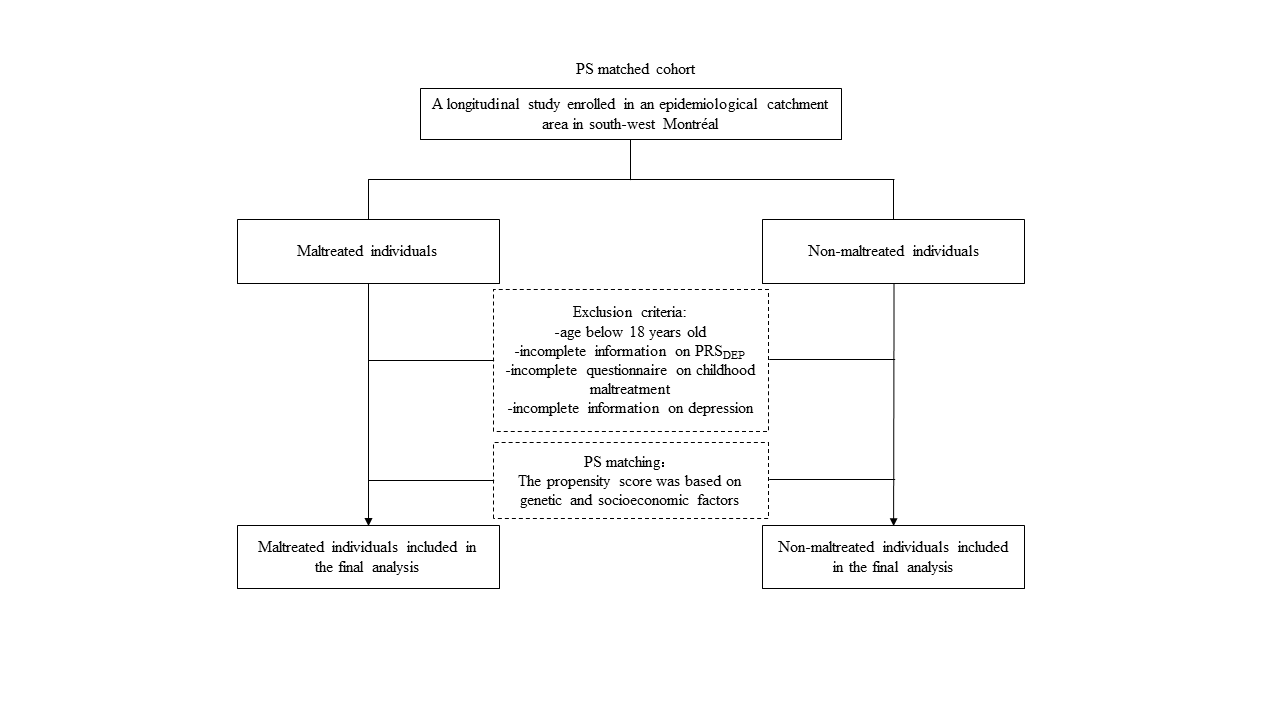


**Figure S1**. Schematic flow diagram of the procedure for propensity score matching.


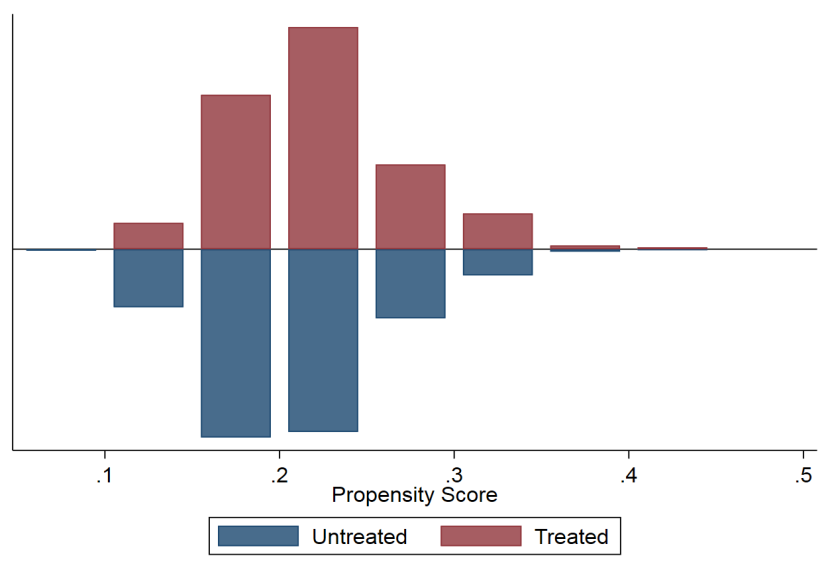

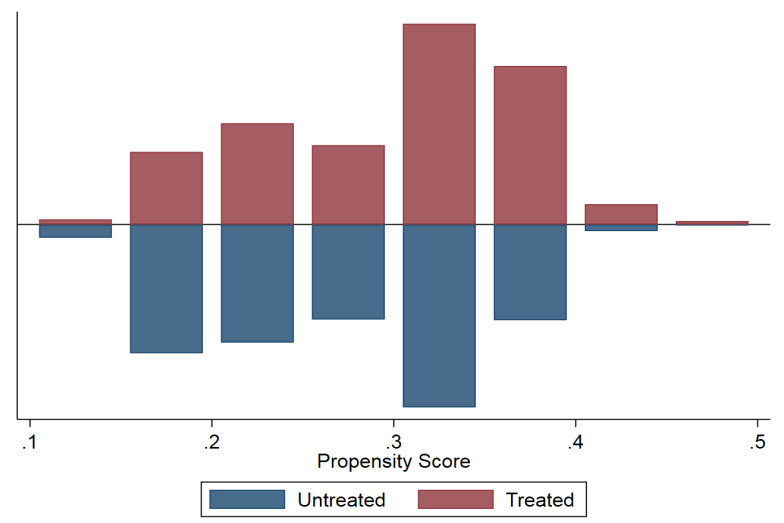


A

C

B


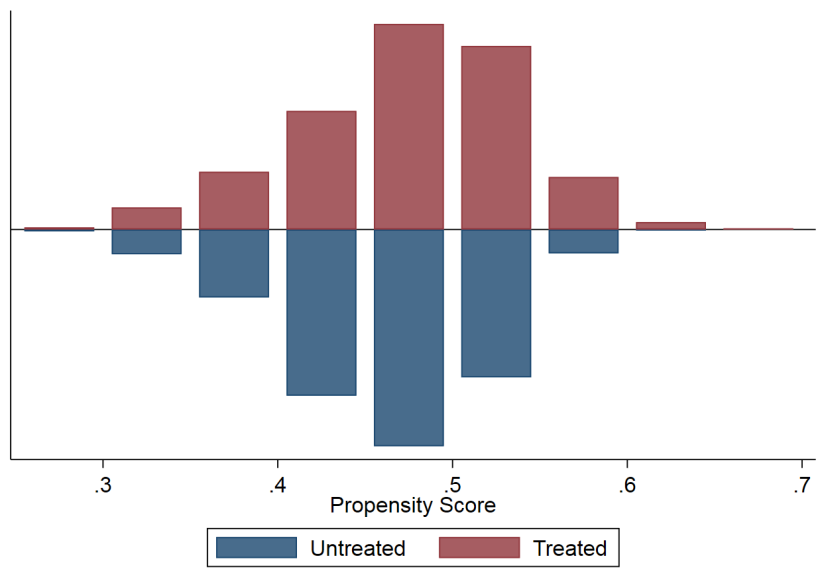

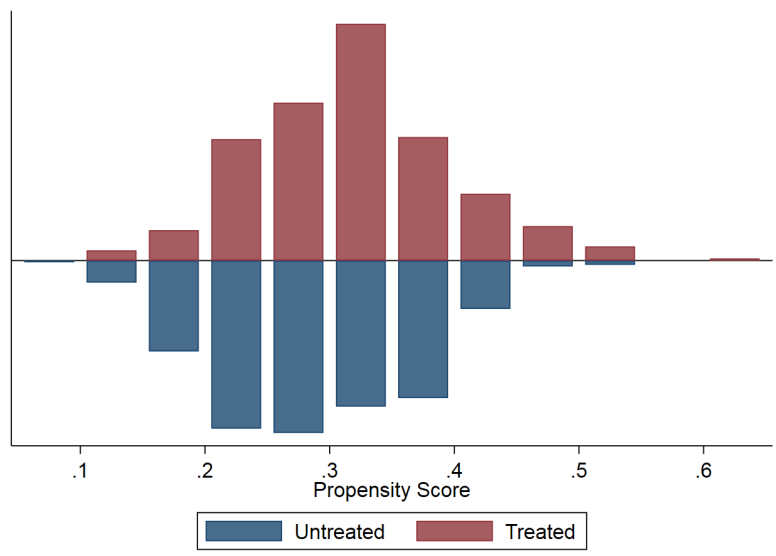


D


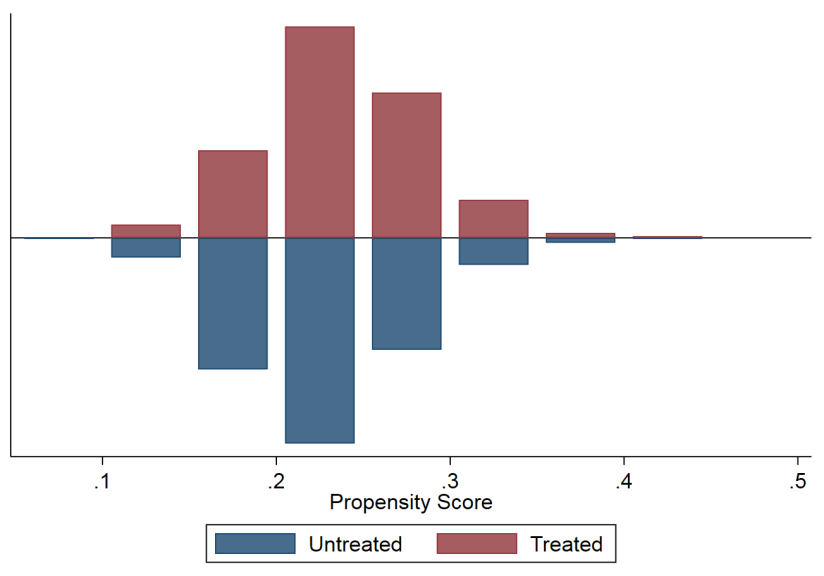


E

**Figure S2**. Propensity score distributions. A. Emotional abuse; B. Physical abuse; C. Sexual abuse; D. Emotional neglect; E. Physical neglect.
